# Dr. Ella Florence Preston

**Published:** 1918-12

**Authors:** 


**Dr. Ella Florence Preston,** Eclectic Institute of N. Y. 1882, died at her home in Dansville, Oct. 15, aged 70.

